# Isolation of *Tibet Orbivirus* from *Culicoides jacobsoni* (Diptera, Ceratopogonidae) in China

**DOI:** 10.1186/s13071-021-04899-9

**Published:** 2021-08-28

**Authors:** Ying Liang Duan, Zhen Xing Yang, Glenn Bellis, Le Li

**Affiliations:** 1 Yunnan Tropical and Subtropical Animal Virus Diseases Laboratory, Yunnan Animal Science and Veterinary Institute, Kunming, Yunnan China; 2 Research Institute for the Environment and Livelihoods, Charles Darwin University, Darwin, NT Australia; 3 Department of Agriculture, Water and the Environment, Darwin, NT Australia

**Keywords:** *Culicoides*, *C*. *jacobsoni*, *C*. *tainanus*, *TibetOrbivirus*, China

## Abstract

**Background:**

*Tibet Orbivirus* (TIBOV) is a recently discovered *Orbivirus* known to infect cattle, Asian buffalo and goats in south-western China. It was first isolated from mosquitoes and subsequently from biting midges (*Culicoides* spp.) in Yunnan, China, indicating that it is an arbovirus. Little is known of its potential to cause disease, but the economic importance of related viruses promoted an investigation of potential *Culicoides* spp. vectors of TIBOV.

**Methods:**

Biting midges were collected approximately once per week between May and December 2020, at a cattle farm in Wulong village, Shizong County, Yunnan Province, China. Approximately 3000 specimens of nine species were subsequently used in attempts to isolate virus, and a further 2000 specimens of six species were tested for the presence of bluetongue virus (BTV) and TIBOV using a RT-qPCR test.

**Results:**

Virus isolation attempts resulted in the isolation of three viruses. One isolate from a pool of *Culicoides* *jacobsoni* was identified as TIBOV, while the other two viruses from *C.* *orientalis* and *C.* *tainanus* remain unidentified but are not BTV or TIBOV. RT-qPCR analysis did not detect BTV in any specimens, but a single pool containing five specimens of *C*. *jacobsoni* and another containing five specimens of *C*. *tainanus* produced PCR quantification cycle (Cq) values of around 28 that may indicate infection with TIBOV.

**Conclusions:**

The isolation of TIBOV from *C*. *jacobsoni* satisfies one criterion required to prove its status as a vector of this virus. This isolation is supported by a low Cq value produced from a different pool of this species in the RT-qPCR test. The low Cq value obtained from a pool of *C*. *tainanus* suggests that this species may also be able to satisfy this criterion. Both of these species are widespread throughout Asia, with *C*. *jacobsoni* extending into the Pacific region, which raises the possibility that TIBOV may be more widespread than is currently known.

**Graphical abstract:**

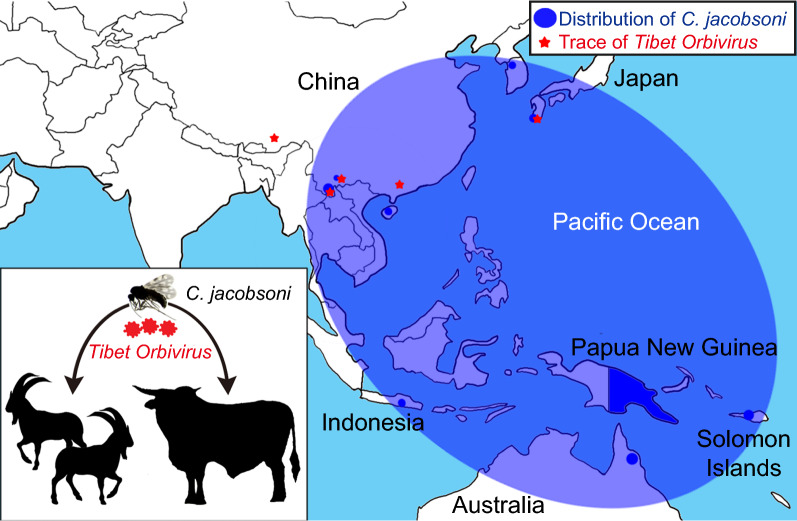

**Supplementary Information:**

The online version contains supplementary material available at 10.1186/s13071-021-04899-9.

## Background

Seven taxonomic families of double-strand (ds)RNA viruses are currently recognized, of which only species of *Reoviridae* are known to infect mammals [1]. Viruses belonging to this family are characterized by possessing multiple dsRNA fragments and double layers of capsids and have been placed into two subfamilies and 15 genera [1, 2]. One of these genera, *Orbivirus*, includes 22 species ratified by the International Committee on Taxonomy of Viruses (2020) [2, 3] and includes the most economically important species in the family, including bluetongue virus (BTV) and African horse sickness virus (AHSV) [4, 5]. It is notable that the economic importance of many of the other species of genus *Orbivirus* remains unknown.

A novel species of *Orbivirus*, *Tibet* *Orbivirus* (TIBOV), was isolated relatively recently, in 2009, from *Anopheles maculatus* in Medog County in the Nyingchi area of Tibet, China, making it the 23rd species in genus *Orbivirus* [6]. However, a virus had been isolated from *Culex tritaeniorhynchus* in 2007, although it was not identified as TIBOV at that time or reported until 2017 [7]; consequently, 2007 is actually the earliest known occurrence of this virus. TIBOV was subsequently isolated from unidentified *Culicoides* from Yunnan Province, China [8, 9], supporting its status as an arbovirus. A limited investigation in Yunnan Province found relatively high antibody prevalence rates in cattle and Asian buffalo and lower rates in goats, indicating that these species are mammalian hosts of this virus [9]. The infection of bovids with TIBOV introduces the possibility that this virus could be a potential pathogen for these or other vertebrate species, as has been shown for other *Orbivirus* such as BTV [1, 10].

The genus *Culicoides* (Diptera: Ceratopogonidae) has been implicated in the transmission of several species of *Orbivirus* [10, 11], strongly suggesting that species in this genus should be considered as possible vectors of TIBOV. At least 40 species of *Culicoides* are currently known to be vectors or potential vectors of pathogens, including at least 50 species of arboviruses [12, 13]. Identification of the vectors is a vital step in understanding the epidemiology of arboviruses, with those causing animal diseases of particular importance. Proven vectors of an arbovirus must satisfy four criteria, two of which are the recovery of virus from wild-caught specimens and an accumulation of field evidence confirming the significant association of the infected arthropods with the appropriate vertebrate population in which disease or infection is occurring [4, 5]. Recently, *Culicoides* *tainanus* and *C*. *jacobsoni*, both of which are common and widespread species of biting midges in Asia, were reported as satisfying these two criteria with respect to BTV in Yunnan Province, China [14, 15], suggesting the potential of these species to be involved with other *Orbivirus*, particularly viruses associated to bovids. In this study, the potential *Culicoides* vectors of TIBOV were studied in Shizong, Yunnan Province, China.

## Methods

### *Culicoides* spp. collection and sorting

Biting midges were collected one night per week for most weeks between May and December 2020 (Table 1), inside a shed containing four penned cattle located in Wulong village, Shizong County, Yunnan Province, China (24°63′N, 104°29′E). A single battery-powered UV light trap (LTS-M02; Wuhan Lucky Star Medical Treatment Technology Co., Wuhan, China) was run from 17:00 h to 09:00 h the following day. Midges were collected directly into phosphate-buffered saline (PBS) (May to November) or 70% ethanol (December) and transported without refrigeration to the laboratory within 24 h of collection. *Culicoides* were sorted by gross morphology and wing pattern [16–18], then parous female specimens without blood meal were washed once in PBS and kept at 4 °C in either PBS containing two antibiotics (100 U/ml ampicillin and 0.1 mg/ml streptomycin; referred to further as PBS + double antibiotics) (Gibco™, Thermo Fisher Scientific, Waltham, MA, USA) for viral isolation or in 75% ethanol for PCR analysis. All species that were collected in sufficient numbers were tested, with a particular focus on *C*. *tainanus* and *C*. *jacobsoni* as they have been recently reported as potential vectors of BTV in China and Asia [14, 15, 19].

**Table 1:** Relative seasonal abundance of *Culicoides tainanus* and *C. jacobsoni* in light traps set in a cattle shed in Shizong, Yunnan Province, China between May and December 2020

Date^a^	Weather^b^	Temperature (°C)	Number of biting midges collected (%)		
			All specimens	*Culicoides tainanus*	*Culicoides jacobsoni*
20 May	Cloudy/sunny	19–29	394	226 (57.4)	0 (0)
25 May	Overcast/light rain	12–23	1330	291 (21.9)	2 (0.2)
05 June	Overcast/light rain	17–28	275	78 (28.4)	0 (0)
09 June	Cloudy/light rain	17–24	1756	644 (36.7)	2 (0.1)
17 June	Overcast	18–25	924	301 (32.6)	9 (1.0)
07 July	Cloudy/light rain	19–27	10	2 (20.0)	0 (0)
14 July	Overcast	17–26	203	61 (30.0)	26 (12.8)
21 July	Cloudy	17–23	282	102 (36.2)	6 (2.1)
28 July	Sunny/light rain	14–25	19	5 (26.3)	7 (36.8)
04 August	Light rain	17–24	1894	487 (25.7)	213 (11.2)
12 August	Overcast/light rain	18–26	416	43 (10.3)	16 (3.8)
08 September	Cloudy/shower	17–24	1847	343 (18.6)	178 (9.6)
15 September	Shower	ND	2057	38 (1.8)	19 (0.9)
22 September	Shower/light rain	ND	954	91 (9.5)	222 (23.3)
28 September	Shower	ND	401	50 (12.5)	93 (23.2)
13 October	Cloudy	ND	245	24 (9.8)	32 (13.1)
20 October	Light rain	ND	1788	674 (37.7)	101 (5.6)
27 October	Cloudy/light rain	ND	1723	601 (34.9)	31 (1.8)
03 November	Light rain	ND	270	65 (24.1)	37 (13.7)
18 November	Cloudy/sunny	ND	507	358 (70.6)	21 (4.1)
02 December	Light rain	ND	461	404 (87.6)	3 (0.7)
11 December	Cloudy	ND	493	389 (78.9)	3 (0.6)
20 December	Sleet	ND	21	18 (85.7)	0 (0)
Total			18,270	5295 (29.0%)	1021 (5.6%)

*ND* the data were not detected

^a^Colllection date

^b^The weather on the dates traps were set and midges collected

### Competitive enzyme-linked immunosorbent assay for cattle blood

Serum samples were collected from the four penned cattle once per week between May and October in 2020. A competitive enzyme-linked immunosorbent assay (C-ELISA) was used to detect BTV antibody in these sera [20].

### Virus isolation

For virus isolation, collected *Culicoides* were placed into pools of no more than 100 conspecific specimens, washed once in 1 ml PBS + double antibiotics (Gibco™, Thermo Fisher Scientific) and suspended in 1 ml minimum essential medium (MEM; Gibco™) + double antibiotics. Pools were homogenized using a multiple functional homogenizer TissueLyser II (Qiagen, Hilden, Germany) for three cycles of 30 pulses per second for 45 s with an interval of 5 s between cycles. Homogenized samples were centrifuged at 12,000 rpm at 4 °C for 20 min, and 400-μl aliquots of supernatant were inoculated onto baby hamster Syrian kidney cell line BHK-21 or *Aedes albopictus* cell line C6/36, respectively. Cells were cultured and monitored as described by Li et al. [6]. The supernatants collected from cultures with a cytopathic effect (CPE) were stored at − 80 °C.

### Pooling of specimens for nucleic acid extraction and testing for the presence of viruses

Pools submitted for nucleic acid extraction comprised specimens belonging to a single species. Specimens were digested either individually (to confirm prescence of cytochrome* c* oxidase subunit 1 [*cox*1]) or in pools of five conspecific specimens (to detect virus). For virus detection, an aliquot of lysate was taken from eight of these pools.

### Nucleic acid extraction

Prepared *Culicoides* specimens were submitted for non-destructive nucleic acid extraction using a procedure described by Duan et al. [14, 15]. Briefly, midges were incubated in 50 μl of lysis buffer from a Genomic DNA Extraction kit (TIANGEN, Beijing, China) at 30 °C for 16 h. The DNA and RNA were extracted together using a MagMAX™-96 Viral RNA Isolation kit following the manufacturer’s directions and a MagMAX™ Express-96 magnetic particle processor (both Ambion Inc., Thermo Fisher Scientific). Nucleic acids were eluted with 50 μl of elution buffer and stored at − 20 °C. A similar procedure was followed to extract RNA from 50 μl of supernatant obtained from BHK-21 or C6/36 cells with CPEs caused by infection with viruses isolated from collected *Culicoides* specimens.

For TIBOV strain identification, a T75 flask of mock (supernatant from regular cell culture used as negative control) or viral isolate-infected BHK-21 cells at 72 h post-infection [hpi]) was scraped and the recovered cells centrifuged at 360 *g* for 5 min. The cell pellets were then transferred to 1.5-ml tubes, suspended in approximately 100 μl supernatant and lysed by freezing and thawing twice, followed by incubation with 25 U of recombinant DNase I (Takara Bio, Osaka, Japan), 50 µg RNase A (Takara Bio) and 100 U of Cryonase™ Cold-active Nuclease (Takara Bio) at 37 °C for 16 h [20]. This process resulted in cellular DNA and RNA being removed while the viral genomes of the virions remained conserved within their capsid. Total RNA was then extracted by a method modified from the common manual method [21]. Briefly, each sample was lysed by 1.2 ml Trizol (Invitrogen®, Thermo Fisher Scientific, Waltham, MA, USA) and incubated at room temperature (RT) for 10 min, then mixed with 220 μl chloroform and incubated at RT for 3 min. The supernatant (about 1 ml per sample) was collected and centrifuged at 13,000 *g* at 4 °C for 10 min, mixed with an equal volume of isopropanol, then incubated at RT for 15 min. RNA was deposited by centrifugation (13 000 *g*, 4 °C, 10 min) and washed once in 1 ml of cold 75% ethanol. Deposited RNA was air dried at RT and suspended in 20 μl of RNase-free water.

### Reverse transcription-quantitative PCR

Specimens from the six most abundant species collected were tested for the presence of TIBOV and BTV by reverse transcription-quantitative PCR (RT-qPCR). First, pools of five conspecific specimens were digested together, then a 10-μl aliquot of lysate was taken from eight of these pools and mixed together to form 80 μl of mixed lysate, representing 40 specimens, for preliminary testing by RT-qPCR. A 2-μl aliquot of RNA was taken from each 80-μl mixed lysate and submitted for RT-qPCR processing using primers and 6-carboxy-fluorescein (FAM) conjugated probes (Table 2) targeting Seg9 of TIBOV, as described by Yang et al. [22]. Samples with a quantification cycle (Cq) value < 40 in the RT-qPCR potentially contain positive samples so these were tested further to ascertain which of the eight pools in the mixture contained the potentially infected insect. A 2-μl aliquot of RNA extracted from each of the eight pools that comprised the potentially positive large pools was processed individually using the same RT-qPCR. A One Step PrimeScript™ RT-PCR kit (Takara Bio) was used to confect the reaction solution and the reaction was performed as described by the manufacturer using a 2-μl RNA sample in a total volume of 20 μl. RT-qPCR was performed on a Fast7500 Realtime PCR system (Applied Biosystems™, Thermo Fisher Scientific) with the following cycling conditions: reverse transcription at 42 °C, 4 min; then denaturation at 92 °C/10 s; followed by denaturation at 92 °C/5 s and annealing-extension at 60 °C/34 s for 40 cycles. Fluorescence was measured at the end of each extension step. Positive controls with a Cq between 23 and 25, and a negative control (water) without signal were run with each batch tested.

**Table 2:** Primers and probes used to target *Tibet Orbivirus* genes

Target	Name of primer or probe^a^	Sequences	Length of PCR products (bp)
Seg4/VP4	TIBOV-Seg4-F	5-TGCGCTATACAGTGCAGAAG	1145
	TIBOV-Seg4-R	5-AATCCGCCACATAAGATCC	
Seg5/NS1	TIBOV-Seg5-F	5-TTGCCACCAGATGCGTATCA	707
	TIBOV-Seg5-R	5-GCTGTYGTAATCAAYGCTTCCA	
Seg9/VP6	TIBOV-Seg9-F	5-AAGAGCGGAAGGAAGAGAG	855
	TIBOV-Seg9-R	5-GCTACGGTCAGGTCTACATC	
Seg9/VP6	TIBOV-YG-S9-F	5-CTACGGAACGAGGAGGGGAT	99
	TIBOV-YG-S9-R	5-CTCGCTGCACATTTCCATCTC	
	TIBOV-S9-Probe	5-ATCAGCTCGTCCTCCTCCCTCTCGT	

TIBOV, *Tibet Orbivirus*

^a^6-Carboxy-fluorescein (FAM) and quenching group BHQ1 were conjugated on the 5’ and 3’ ends of the probe, respectively

The presence of BTV RNA was tested in the same pools of insect lysate using a pan BTV serotype RT-qPCR method [15] with primers BTVF-MH and BTVR-MH and probe BTVP-MH [23]. Properly diluted positive controls with a Cq of approximately 25 and a negative control (water) without signal were run with each batch tested.

### Reverse transcription for viral RNA

The cDNA of viral RNA was synthesized using the kit of the SuperScript® III First-Strand Synthesis System (Invitrogen™, Thermo Fisher Scientific) according to the manufacturer’s instructions. Briefly, 8 µl of RNA was mixed with 1 µl dNTPs and 1 µl random primers provided by the kit, then denatured at 95 °C for 1 min followed by quick cooling on ice. Buffers and reverse transcriptase (RTase) were added, and a total of 20 μl cDNA per sample was produced by a RT process: incubation at 25 °C/10 min, 45 °C/30 min and 85 ℃/5 min; then temporary storage at 4 °C. Following the temporary storage, 1 μl ribonuclease H was added followed by incubation at 37 °C for 10 min to delete the RNA.

### PCR for TIBOV

Primers targeting three separate TIBOV genes (Table 2) were used to amplify viral genes. For each test, 0.5 μl of cDNA was added to 9.5 µl PCR solution which was confected with primers and Taq-PCR premix (TIANGEN). For sequencing, 3 µl of cDNA was used in a 50-μl reaction system confected with primers and PrimeSTAR® HS premix (Takara Bio). The PCR cycling program consisted of: denaturation at 95 ℃, 2 min; denaturation at 95 ℃/10 s, annealing at 54 ℃/10 s, extension at 72 ℃/1 min (Taq) or at 68 ℃/1 min (PrimeSTAR) for 30 cycles; and a final extension at 72 ℃ or 68 ℃ for 1 min, with storage at 4 ℃.

### PCR for *cox*1 amplification

All specimens submitted for *cox*1 amplification were processed individually. A 5.5-µl aliquot of nucleic acid from each specimen was added to a 30-µl reaction volume containing PrimeSTAR HS DNA Polymerase, a high-fidelity DNA polymerase (Takara Bio), and amplified according to Duan et al. [14, 15].

The DNA products were sent to Kunming Shuoqing Biological Technology Company (Kunming, China) for sequencing. *Cox*1 sequences were queried for best matched species in the National Center for Biotechnology Information (NCBI) and Barcode of Life Data System (BOLD) databases.

### Electrophoresis

A 2.5-µl aliquot of DNA marker AL5000 (Aidlab, Aidlab Biotechnologies Co., Ltd, Beijing, China), a 10-µl aliquot of BHK-21 RNA or viral RNA and all of the Taq PCR products were loaded in 1% agarose gel with nucleic acid dye Goldview II (Solarbio, Beijing, China). DNA fragments were separated by electrophoresis at 100 V for 1 h. Fluorescent bands were screened by a Gel Doc™ XR+ System with Image Lab™ software (Bio-Rad, Hercules, CA, USA) and used for testing the TIBOV PCR products in virus isolates.

### TIBOV sequence analysis

Amino acid sequences of NS1 proteins and VP6 proteins of 31 representative viral strains belonging to six species of *Orbivirus* were downloaded from the NCBI database. Sequences were aligned using the Muscle algorithm, truncated and then phylogenetic trees were constructed by the maximum likelihood (ML) algorithm with Poisson model (bootstrap = 1000) using MEGA-X software [24].

## Results

### *Culicoides* collections

**Fig. 1:**
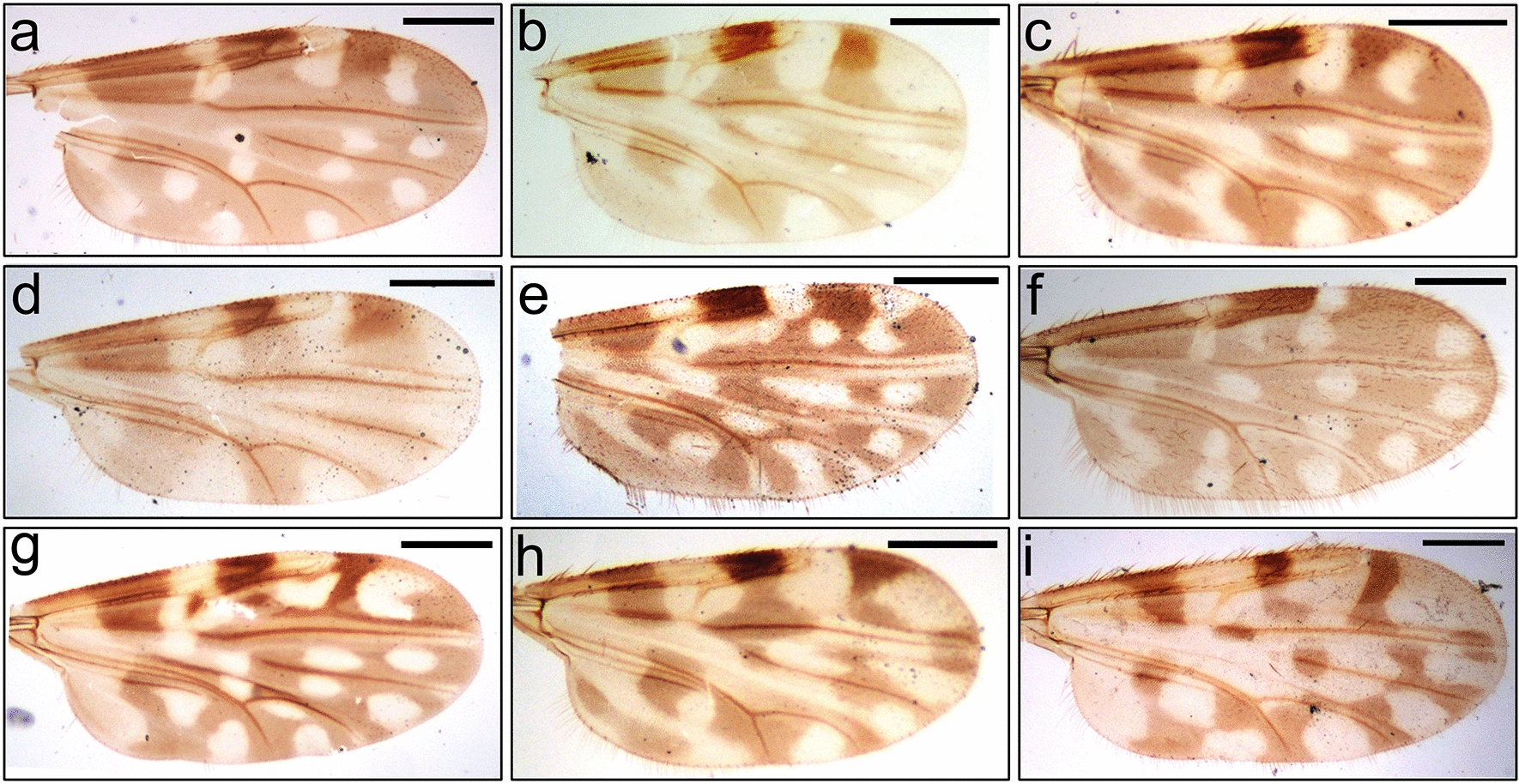
Wing patterns of the major *Culicoides* spp. identified in this study. **a** *C. sumatrae*, **b** *C. tainanus*, **c** *C. jacobsoni*, **d** *Culicoides* (*Trithecoides*) spp. with a yellow scutum, **e** *C. oxystoma*, **f** *C. arakawai*, **g** *C. insignipennis*, **h** *C. orientalis*, **i** *C. liui*. All wings come from female specimens. Scale bar: 250 µm

More than 18000 *Culicoides*, including approximately 5300 *C*. *tainanus* and 1000 *C*. *jacobsoni*, were retrieved from 23 collections (Table 1). At least 23 *Culicoides* species were identified. Representative wing patterns of the nine species used in isolation or qPCR testing are shown in Fig. 1.

Full data on the relative abundance and seasonality of all species collected will be published elsewhere. However, *C*. *sumatrae* was the most predominant species (32.7%) in the latter half of 2020 while *C*. *tainanus* was the second most dominant species and appeared in all of the collections. The relative proportion of *C*. *tainanus* in each batch was 1.8–87.6%, with an average abundance of 29.0% during the latter half of 2020, reaching peak abundance (70.6-87.6%) in the winter (Table 1). *Culicoides jacobsoni*, however, mainly appeared in the summer and showed peak abundance between August and October. The relative proportion of *C*. *jacobsoni* in each collection was 0–36.8% with an average value of 5.6% (Table 1).

### TIBOV and BTV RT-qPCR testing of *Culicoides*

A total of 2000 biting midges were processed in 400 pools, with each pool containing five specimens of a single species, and tested for the presence of TIBOV or BTV by RT-qPCR (Table 3). Positive reactions to TIBOV RNA were observed in 14 of these pools by RT-qPCR analyses targeting TIBOV Seg10. Most of these reactions had a Cq > 30; however one pool of *C*. *tainanus* had a Cq of 28.0 and one pool of *C*. *jacobsoni* had a Cq of 28.6 (Table 3; Additional file 1: Figure S1). No pools showed any reaction to the BTV test. No evidence associating the most dominant species *C*. *sumatrae* with BTV or TIBOV was found in this study (Table 3).

**Table 3:** Screening of *Culicoides* midges from Shizong County, Yunnan Province, China, for the presence of TIBOV by reverse transcription-quantitiative PCR

Species	Midges tested by RT-qPCR (*n*)	Pools tested by RT-qPCR (*n*)			
		Total	Cq < 30	30 ≤ Cq < 35	Cq > 35
*C. tainanus*	800	160	1	2	6
*C. jacobsoni*	400	80	1	0	2
*C. sumatrae*	300	60	0	0	0
*C. oxystoma*	300	60	0	0	0
*Culicoides* (*Trithecoides*) spp.^a^	120	24	0	0	1
*C. insignipennis*	80	16	0	1	0
Total	2000	400	2	3	9

Cq, PCR quantification cycle; RT-qPCR, reverse transcription-quantitiative PCR

^a^*Culicoides* (*Trithecoides*) spp. refers to specimens belonging to this subgenus that have a yellow scutum

*Cox*1 fragments were successfully amplified from the nucleic acid samples of the two TIBOV-positive pools (*C*. *tainanus* and *C*. *jacobsoni*) and from six individual *C*. *tainanus* specimens and six individual *C*. *jacobsoni* specimens collected from the same location at Shizong. The *cox*1 sequences from the *C*. *tainanus* pool and from the *C*. *jacobsoni* pool were 100% similar with their six respective conspecific specimens. Sequences from one *C*. *tainanus* (YN/2020/T6) and one *C*. *jacobsoni* (YN/2020/J6) have been deposited in GenBank (Table 4). Comparison of sequence data with publicly available data matched the two pools of *Culicoides* infected by TIBOV to specimens identified as *C*. *tainanus* and *C*. *jacobsoni*, respectively. The closest matches on GenBank were two *C*. *tainanus* from Taiwan and one *C*. *jacobsoni* from South Korea, respectively (Table 4). The BOLD database placed our potentially infected *C*. *tainanus* (MW585344) to the Barcode Index Number (BIN) BOLD:AAI9872 containing specimens from Taiwan and Japan; while our *C*. *jacobsoni* (MW585343) belongs to BIN BOLD:AAI9869 (data not shown) which contains specimens from eight countries or regions (Malaysia, Solomon Islands, China, Reunion, Japan, Vietnam, Indonesia and Papua New Guinea), as well as from South Korea based on the 100% similarity of *cox*1 between the South Korea specimen (KF297817.1) and our specimen (MW585343) (Table 4) [15, 25].

**Table 4:** Genetic similarity to sequence data in GenBank and Barcode of Life Data System of pools of *C. tainanus* and *C. jacobsoni* which produced low Cq reactions to a RT-qPCR test for TIBOV

*Culicoides* species	GenBank			BOLD	
	Accession no.	Accession no.	Similarity	BIN no.	Similarity
*C. jacobsoni*	MW585343	KF297817.1	100%	AAI9869	100%
*C. tainanus*	MW585344	MK760246.1	99.78%	(Unpublished)^a^	100%

BOLD, Barcode of Life Data System; NCBI, National Center for Biotechnology Information

^a^The best matched BIN for our *C. tainanus* was unpublished in BOLD

### Isolation of viruses from *Culicoides*

Virus isolation was attempted on 41 pools of midges from eight morphologically identified species and from mixed species of *Culicoides* belonging to the subgenus *Trithecoides* which were unable to be identified (Table 5). Three viruses were isolated from separate pools of *C*. *tainanus*, *C*. *orientalis* and *C*. *jacobsoni* while no virus was isolated from any of the other species (Table 5). These isolates were designated as YNV/01-1, YNV/03-2 and YNV/17-14, respectively, and submitted for molecular analysis to identify the viruses.

**Table 5:** Virus isolation attempts from *Culicoides* collected from Shizong County in Yunnan Province, China

Species	Approximate number used for testing	Pools^a^	Cells with cytopathic effect	Isolate designation	Virus
*C. sumatrae*	1050	0/12			
*C. tainanus*	820	1/9	C6/36	YNV/01-1	Unknown
*Culicoides* (*Trithecoides*) spp.^b^	350	0/4			
*C. orientalis*	300	1/4	BHK-21, C6/36	YNV/03-2	Unknown
*C. arakawai*	200	0/3			
*C. jacobsoni*	200	1/2	BHK-21, C6/36	YNV/17-14	TIBOV
*C. insignipennis*	180	0/4			
*C. oxystoma*	40	0/2			
*C. liui*	20	0/1			
Total	3160	3/41			

^a^Values in this column are presented as positive number of pools/total number of pools

^b^*Culicoides* (*Trithecoides*) spp*.* refers to specimens belonging to this subgenus with a yellow scutum

**Fig. 2:**
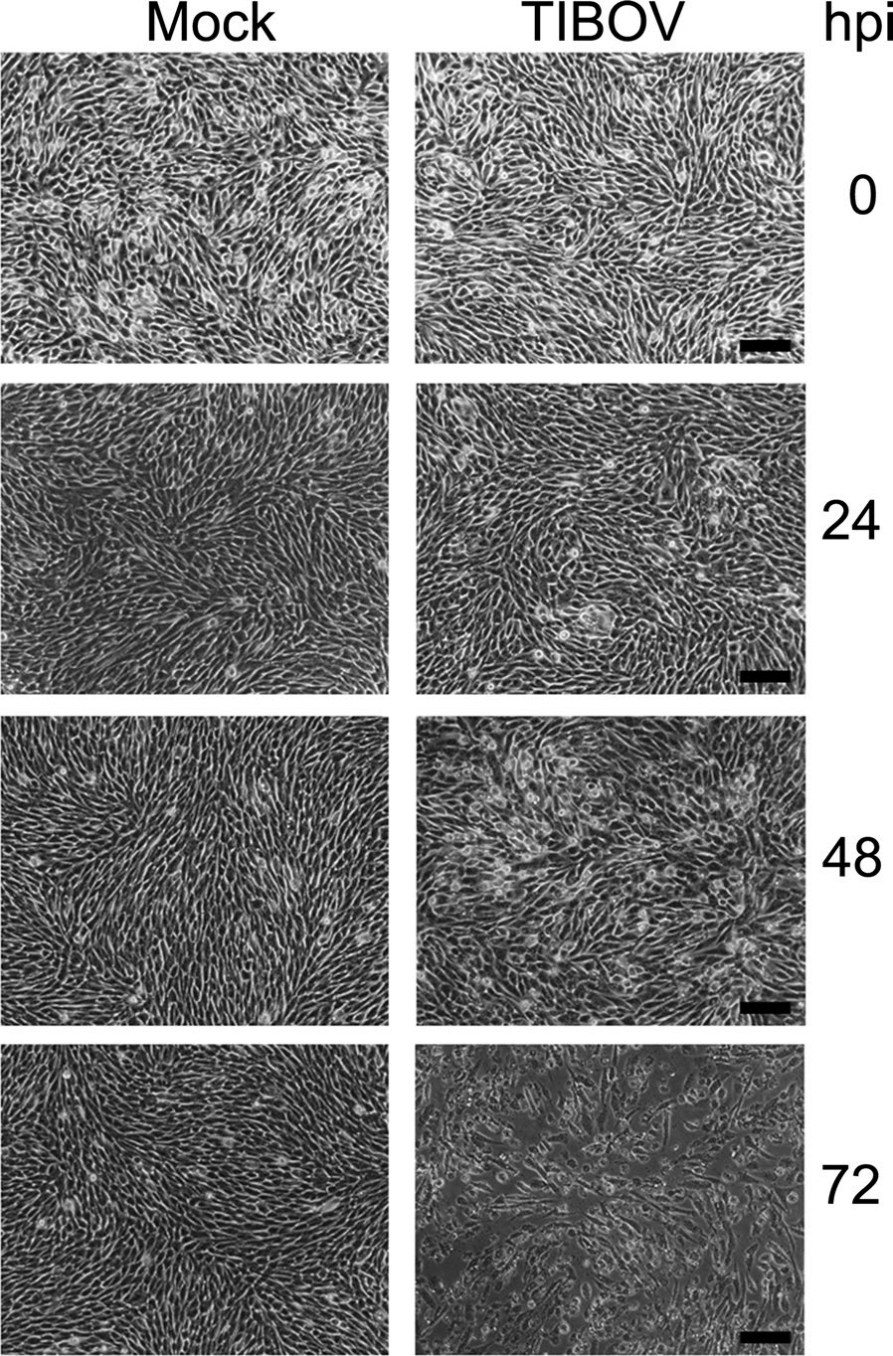
Cytopathic effects caused by infection with *Tibet Orbivirus* (TIBOV) isolate.* Mock* Non-virus medium inoculated baby hamster Syrian kidney cell line BHK-21, * TIBOV* *Tibet Orbivirus* viral strain YNV/17-14-infected BHK-21 cells,* hpi* hours post-infection. Scale bar: 100 µm

BHK cells inoculated with isolate YNV/17-14 from *C*. *jacobsoni* became intumescent and showed suspected CPEs at 24 h post-infection (hpi), with obvious CPEs at 48 hpi; there was almost complete cell necrosis or apoptosis at 72 hpi (Fig. 2). This observation suggests that most cells were infected by the TIBOV but that there was no obvious CPE until 48 hpi.

### Identification of TIBOV isolate

The RT-qPCR analyses failed to amplify BTV or TIBOV RNA from the viruses YNV/01-1 and YNV/03-2 isolated from *C*. *tainanus* and *C*. *orientalis*, and further work is needed to identify these isolates. The virus isolated in BHK cells from the single pool of *C*. *jacobsoni*, YNV/17-14 (Additional file 2: Table S1), however, produced a positive reaction to the TIBOV RT-qPCR.

**Fig. 3:**
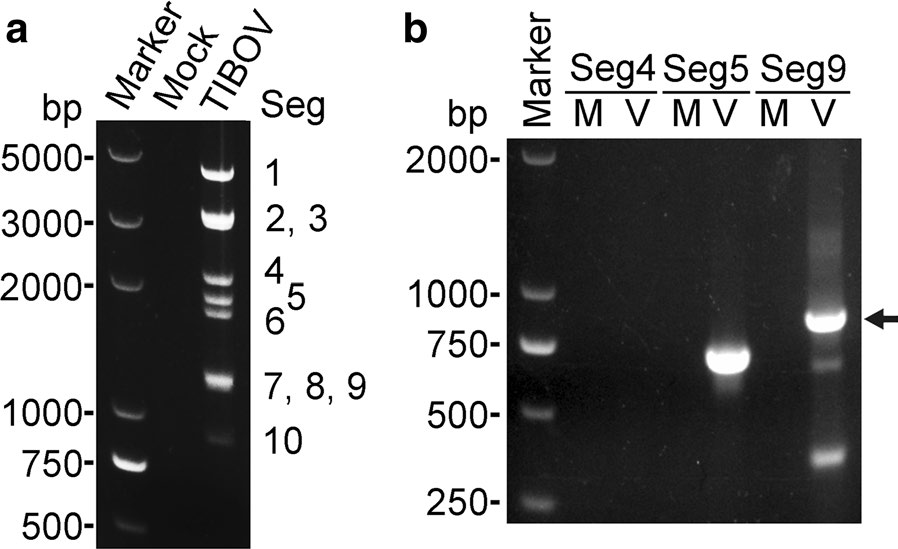
Genome and PCR products of TIBOV isolate YNV/17-14. **a** Total RNA without cellular nucleic acids extracted from Mock (medium) and YNV/17-14-infected BHK-21 cells. **b** The reverse transcription-quantitative PCR products of Seg4, Seg5 and Seg9 of TIBOV amplified from total RNA. *M* mock infected, *V* TIBOV infected. Arrow identifies the target product of Seg9

Electrophoresis of isolate YNV/17-14 revealed ten discrete segments with a 3-3-3-1 pattern consistent with BTV and TIBOV (Fig. 3a). Three pairs of primers specific for TIBOV genes successfully amplified Seg5 and Seg9 of isolate YNV/17-14, confirming it as a strain of TIBOV. The failure to amplify Seg4 might be caused by intraspecific sequence differences between the primers and template (Fig. 3b).

**Fig. 4:**
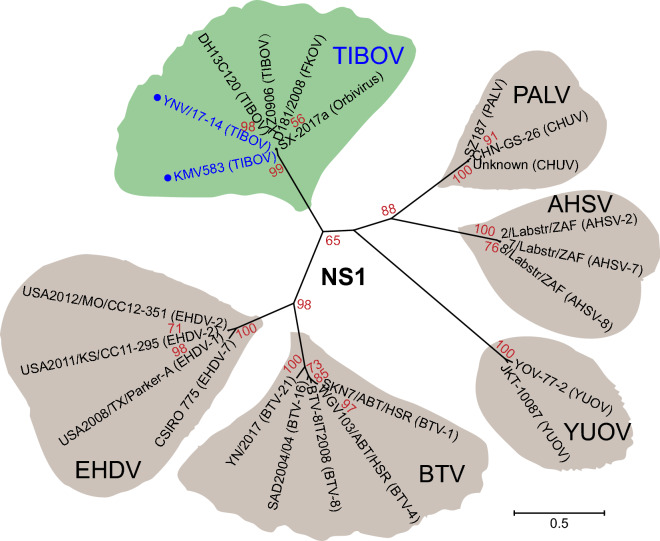
Phylogenetic tree of representative *Orbivirus* based on non-structural protein 1 (NS1) amino acids. The tree is constructed using the maximum likelihood algorithm with Poisson model (bootstrap = 1000); bootstrap values < 50% are omitted. For each virus, strain name, species and serotype are provided. Detailed information of each virus is listed in Additional file 2: Table S1

The PCR products of Seg5 and Seg9 from isolate YNV/17-14 and from the suspected positive pool of *C*. *tainanus* midges labeled KMV583 (Table 3; Additional file 1: Figure S1) were sequenced and found to match TIBOV genes registered in GenBank. These four sequences of TIBOV YNV/17-14 (MW436463, MW436464) and KMV583 (MW465962, MW465963) have been lodged with GenBank (Additional file 2: Table S1). A phylogenetic tree created from non-structural protein 1 (NS1) proteins (usually encoded by Seg5) from 23 viruses belonging to six species of *Orbivirus* showed that our two virus strains were clustered with a group of four strains of TIBOV reported previously, and the branch distances suggested that TIBOV is closely related to epizootic haemorrhagic disease virus (EHDV) and BTV, but distantly related to *Yunnan Orbivirus* (YUOV) (Fig. 4). A phylogenetic tree based on VP6 proteins (usually encoded by Seg9) produced a similar result (Additional file 3: Figure S2). All sequences used in our phylogenetic trees were downloaded from GenBank, and their accession numbers are listed in Additional file 2: Table S1.

### C-ELISA test for cattle sera

All the serum samples were negative for BTV antibodies based on the results from the C-ELISA. TIBOV antibodies could not be detected in cattle sera because the reagents required were not available.

## Discussion

*Tibet Orbivirus* (TIBOV), a novel *Orbivirus*, was discovered, isolated, completely sequenced and identified relatively recently [6–9, 26], but has yet to be registered by the International Committee on Taxonomy of Viruses (2020) [2]. So far, the virus is only known from China (Additional file 2: Table S1) [6–9, 26, 27] and Japan [28], and its vertebrate hosts are likely to include cattle, Asian buffalo and goats [9]. There is currently no information on serotypes or genotypes for TIBOV, although limited phylogenetic analysis suggests that multiple serotypes or genotypes do exist (Fig. 4; Additional file 3: Figure S2) [7, 26].

*Culicoides* spp. are vectors of at least 50 species of arbovirus [12, 13], including EHDV, AHSV and Palyam virus (PALV) [8, 12, 13, 29, 30], and thus an association between *Culicoides* and TIBOV is not surprising. The isolation of TIBOV from *C*. *jacobsoni* provides evidence that this species satisfies one of the criteria proving it to be a vector of TIBOV. *Culicoides jacobsoni* was one of the predominant species in the collections made during the summer in the cattle shed at Shizong, which supports its potential importance as a vector of this virus although more extensive studies on both the seasonal prevalence of TIBOV and the relative abundance of species of *Culicoides* are warranted to support this observation at Shizong. The taxonomy of *C*. *jacobsoni* is complex, with several potential cryptic species reported by Gopurenko et al. [31]; however, the haplotype collected at Shizong belongs to a distinct DNA barcode cluster (BIN BOLD:AAI9869) that is widespread in Asia and extends into the Pacific. Regional areas where *C*. *jacobsoni* (particularly strain BOLD:AAI9869) overlap with host mammals should be monitored for the presence of TIBOV to determine if this virus is present throughout the insect’s known distribution. If so, this may lend further support to the labeling of *C*. *jacobsoni* as a vector of TIBOV.

Seasonal abundance data suggest that *C*. *tainanus* populations peak during colder times of the year, a finding which is consistent with the largely temperate distribution of this midge species in Asia [14, 16, 17]. However, *C. jacobsoni* appears to prefer warmer weather, which is also consistent with the known distribution of this species in southern Asia and the Pacific [15–17, 31].

Traditionally, evidence of infection of *Culicoides* with orbiviruses was provided by the isolation of virus in the laboratory. However, keeping viruses viable in field-collected specimens is laborious as they are sensitive to any number of factors likely to occur between collection of the specimens and use in virus isolation techniques, such as overheating of samples, delays in transporting specimens and contamination with non-specific bacteria or fungi. Recent advances in PCR technology has allowed field-collected specimens preserved in ethanol to be screened for the presence of virus using the Cq value determined in RT-PCR analyses to distinguish specific and non-specific reactions, thereby allowing reliable interpretation of results [14, 15, 32–36]. Calibration of the test, however, requires an assessment of the threshold Cq value for each virus to enable insects to be distinguished in terms of those in which virus has replicated and those which either retain residual virus from an infected blood meal or have a non-specific reaction in the test. Thus far this calibration has only been conducted using BTV infection in *Culicoides* [32, 34–37], which has allowed for the identification of novel potential vector species of BTV in China [14, 15]. Indeed, the bimodal pattern of reactions observed with field-collected specimens reported by Duan et al. [14, 15], where reactions were observed with Cq values either < 25 or > 30, provides support that the laboratory-based results of Van Der Saag et al. [32] and Veronesi et al. [37] are applicable to field-collected midges. Extrapolation of this result to other viruses is difficult as the level of viremia in the insect may vary, which could alter the Cq value on which determination of infection of the insect is based. Unfortunately, the RT-qPCR assays used for TIBOV have not yet been calibrated to allow reliable identification of insects in which virus has replicated; however, a bimodal pattern of Cq values was observed in this study, with two reactions showing a Cq value of around 28 and the remainder showing a Cq value of > 32.8 (Table 3; Additional file 1: Figure S1). The late Cq emergence here is unlikely to be caused by the formation of primer-dimer because the primers and probe for TIBOV RT-qPCR were designed well. Temporary conservation of midges in PBS within 48 h before sorting may be one of factors that resulted in relatively high Cq values of the positive samples for TIBOV detection in this study, since all of the midges used for BTV detection in our previous reports were kept in 70–90% ethanol all of the time [14, 15]. The fact that one of these reactions with a low Cq value was obtained from a small pool of *C*. *jacobsoni*, a species from which viable virus was also isolated, suggests that a Cq value < 29 may possibly indicate that specimens were infected with virus. Further support for the potentially positive result from the RT-qPCR lies in the successful amplification of seg5 and seg9 from the PCR product obtained from the pool of *C*. *tainanus* and the matching of these gene sequences to isolates of TIBOV. If the use of RT-qPCR to detect infected insects is validated in laboratory studies, then this technique provides evidence that TIBOV has also been detected in field-collected specimens of *C*. *tainanus*, thus satisfying one of the criteria proving this species to be a vector of TIBOV. Should this haplotype of this species be proven to be a vector of TIBOV, then its wide distribution, including Taiwan and Japan, suggests that TIBOV should have a similarly wide distribution.

No evidence of infection with BTV was detected from the *Culicoides* in this study, which was supported by the negative result of BTV antibodies in sampled cattle. This lack of BTV infection may be caused by the relatively low livestock density in the farm studied, although Shizong County is a prevalent area of BTV.

## Conclusions

The detection and isolation of TIBOV in *C*. *jacobsoni* provides the first evidence of the vector status of this midge species for TIBOV and suggests that TIBOV might be more widely distributed than currently known. RT-qPCR results for *C*. *tainanus* may additionally have detected TIBOV in this species, but more work is required to validate this test. Once validated, this test will prove a useful tool in future studies of the ecology of this virus.

## Supplementary Information

**Additional file 1: Figure S1.***Culicoides* (*Trithecoides*) spp. refers to specimens belonging to this subgenus with a yellow scutum. Cq values from RT-qPCR tests for TIBOV from specimens referable to four species of *Culicoides*. Each point represents the Cq value of a pool composed of five conspecific specimens. Pools with Cq values < 30 are labeled red.

**Additional file 2: Table S1.** Amino acid sequences of orbiviruses used in phylogenetic analysis.

**Additional file 3: Figure S2.** Phylogenetic tree of representative *Orbivirus* based on VP6 amino acids. The tree is constructed by the ML algorithm with Poisson model (bootstrap = 1000); bootstrap values < 50% are omitted. For each virus, strain name, species and serotype are provided. Detailed information of each virus is listed in Additional file 2: Table S1.

## Data Availability

Data supporting the conclusions of this article are provided within the article. Raw data are available from the corresponding author upon request. The newly generated sequences were submitted to the GenBank database under the accession numbers: *cox*1 sequences (MW585343 and MW585344); TIBOV Seg5 (MW436463, MW465962) and Seg9 (MW436464, MW465963).

## Abbreviations

AHSV: African horse sickness virus
BIN: Barcode Index Number
BTV: Bluetongue virus
BLAST: Basic local alignment search tool
BOLD: Barcode of Life Data System
*cox*1: Cytochrome* c* oxidase subunit 1
CPE: Cytopathic effect
Cq: Quantification cycle
EEV: Equine encephalosis virus
EHDV: Epizootic haemorrhagic disease virus
hpi: Hours post-infection
MEM: Minimum essential medium
ML: Maximum likelihood
NCBI: National Center for Biotechnology Information
NCS: Newborn calf serum
NS1: Non-structural protein 1
PALV: Palyam virus
RT-qPCR: Reverse transcription quantitative PCR
TIBOV: *Tibet* *Orbivirus*
VP6: Viral protein 6
YUOV: *Yunnan* *Orbivirus*
