# Supplementary material for: Isolation of Tibet Orbivirus from Culicoides jacobsoni (Diptera, Ceratopogonidae) in China
Source: Parasit Vectors. 2021 Aug 28;14:432. doi: 10.1186/s13071-021-04899-9 (PMC8401062; doi:10.1186/s13071-021-04899-9)
Supplement: Supplementary file 2 — Additional file 2: Table S1. Amino acid sequences of orbiviruses used in phylogenetic analysis. [file 13071_2021_4899_MOESM2_ESM.docx]

**Additional file 2: Table S1.** Amino acid sequences of orbiviruses used in phylogenetic analysis

| **Species of *Orbivirus* and strain/isolate** | **Serotype** | **Origin** | |  | **GenBank accession/protein number** | |
| --- | --- | --- | --- | --- | --- | --- |
|  |  | **Country** | **Year** |  | **NS1 protein** | **VP6 protein** |
| **African horse sickness virus** |  |  |  |  |  |  |
| 2/Labstr/ZAF/1998/OBP-252.1 | AHSV-2 | South Africa | 1998 |  | ALG63770.1 | ALG63768.1 |
| 7/Labstr/ZAF/1998/OBP-252.1 | AHSV-7 | South Africa | 1998 |  | ALG63790.1 | ALG63788.1 |
| 8/Labstr/ZAF/1998/OBP-252.1 | AHSV-8 | South Africa | 1998 |  | ALG63800.1 | ALG63798.1 |
| **Bluetongue virus** |  |  |  |  |  |  |
| SKN7/ABT/HSR | BTV-1 | India | 2007 |  | AGW27472.1 | AGW27476.1 |
| WGV103/ABT/HSR | BTV-4 | India | 2008 |  | AIK27563.1 | AIK27567.1 |
| BTV-8IT2008 | BTV-8 | Italy | 2008 |  | AIG14340.1 | AIG14344.1 |
| SAD2004/04 | BTV-16 | Italy | 2004 |  | CAO79535.1 | None |
| YN/2017 | BTV-21 | China | 2017 |  | QDK54957.1 | QDK54961.1 |
| **Epizootic haemorrhagic disease virus** |  |  |  |  |  |  |
| EHD1/USA2008/TX/Parker-A | EHDV-1 | USA | 2008 |  | ALX38652.1 | ALX38560.1 |
| EHD1/USA2010/AL/E-10-4296 | EHDV-1 | USA | 2010 |  | None | ALX38559.1 |
| CC 304-06 | EHDV-2 | USA | 2006 |  | None | AEM06335.1 |
| EHD2/USA2011/KS/CC11-295 | EHDV-2 | USA | 2011 |  | ALX38637.1 | ALX38548.1 |
| EHD2/USA2012/MO/CC12-351 | EHDV-2 | USA | 2012 |  | ALX38636.1 | ALX38547.1 |
| CSIRO 775 | EHDV-7 | Australia | 1981 |  | CAN89143.1 | None |
| **Palyam subgroup virus** |  |  |  |  |  |  |
| (anonymity)^a^ | CHUV | Japan | 1985 |  | BAA76552.1 | BAA76551.1 |
| CHN-GS-26 | CHUV | China | 2016 |  | QCD25268.1 | QCD25277.1 |
| SZ187 |  | China | 2012 |  | ALW83182.1 | ALW83186.1 |
| **Tibet Orbivirus** |  |  |  |  |  |  |
| YNV/17-14 (new submission) |  |  | 2020 |  | (MW436463)^b^ | (MW436464)^b^ |
| KMV583 (new submission) |  |  | 2020 |  | (MW465962)^b^ | (MW465963)^b^ |
| D181/2008 | FKOV | China | 2008 |  | AKV89258.1 | AKV89262.1 |
| SX-2017a |  | China | 2007 |  | AQM73715.1 | AQM73719.1 |
| XZ0906 |  | China | 2009 |  | AHE77365.1 | AHE77369.1 |
| DH13C120 |  | China | 2013 |  | APT68078.1 | APT68081.1 |
| **Yunnan Orbivirus** |  |  |  |  |  |  |
| JKT-10087 |  | Indonesia | 1981 |  | QGU18505.1 | QGU18503.1 |
| JKT-8650 |  | Indonesia | 1981 |  | None | QGU18496.1 |
| YOV-77-2 |  | China |  |  | YP_443929.1 | YP_443933.1 |
| Rioja |  | Peru |  |  | None | ACO53605.1 |

^a^ Name not provided in GenBank submission. ^b^ These numbers are access numbers of genes, since the protein IDs are not created by NCBI yet.
